# P01-034 – Cancer in FMF: a population based study Israel

**DOI:** 10.1186/1546-0096-11-S1-A38

**Published:** 2013-11-08

**Authors:** R Brenner, S Kivity, Y Shinar, I liphshitz, E Ben-Chetrit, A Livneh, B Zvi

**Affiliations:** 1 Cancer Unit, Edith Wolfson Medical Center, Holon, Tel Aviv, Israel; 2 Rheumatology Unit , Zavladovich center of autoimmune disease, Israel; 3 Department of Medicine A and C, Sheba Medical Center, Ramat-Gan, Israel; 4 Heller Institute of Medical Research, Sheba Medical Center, Ramat-Gan, Israel; 5 Israeli National Cancer Registry, Ministry of Health, Israel; 6 Department of Medicine A, Hadassah-Hebrew University and Medical Center, Jerusalem, Israel; 7 Department of Medicine F, Sheba Medical Center, Ramat-Gan, Israel; 8 Rheumatology Unit at Zavladovich center of autoimmune disease, Heller Institute of Medical Research , Department of Medicine F. , Sheba Medical Center, Tel Hashomer, Ramat Gan, Israel

## Introduction

Recurrent or persistent inflammation, featuring Familial Mediterranean Fever (FMF), may induce, promote, or influence susceptibility to carcinogenesis. However, the association between FMF and malignancy was rarely described before.

## Objectives

To assess the prevalence of malignancy in FMF.

## Methods

Demographic data of FMF patients, followed in the national FMF center at Sheba medical center (n=8352) and Hadassah Medical Center (n=1083) were obtained from FMF patient hospital registries. The prevalence of cancer in the general population, and in the study registries was attained from the cancer registry of Israel and analyzed according to age, origin, and cancer type. The Standardized **incidence** rates (SIR) of the different cancers in FMF patients were calculated and compared to the cancer SIR of the parallel Israeli ethnic population.

## Results

Of 9435 FMF patients (4696 men, 4739 female), 363 developed cancer during the years 1970- 2011. FMF female patients developed significantly more lymphoma (Hodgkin and non-Hodgkin) and in-situ cervical cancer than the matched general population, SIR 2.07 (95% CI 1.12-2.99) and 1.86 (95% CI 1.20-2.51), respectively. In contrast, male and female with FMF had a lower gastrointestinal cancer incidence, SIR 0.68 (95% CI 0.42-0.95) and 0.63 (95% CI 0.36-0.90).

## Conclusion

The risk for lymphoma and in-situ cervical cancer is increased about twice in FMF. Understanding the underlying mechanism (inflammation? Colchicine? Genetic predisposition?) may improve patient prognosis.

## Disclosure of interest

None declared.

